# The Medical News Pocket Formulary for 1899

**Published:** 1899-09

**Authors:** 


THE MEDICAL NEWS POCKET FORMULARY FOR 1899. Containing 1,600 prescriptions representing the latest and most approved methods of administering remedial agents. By E. QUIN THORNTON, M.D., Demonstrator of Therapeutics, Pharmacy and Materia Medica in the Jefferson Medical College, Philadelphia. In one wallet-shaped volume, strongly bound in leather, with pocket and pencil. Philadelphia and New York: Lea Brothers & Co. 1899.

One of the handiest little volumes issued this year is the Medical News Pocket Formulary, by E. Quin Thornton, M. D.

The use of a pocket or any other kind of a formulary as a regular practice is not good medicine, but the fact remains, nevertheless, that these little volumes are valuable adjuncts to a practice. They represent the collective experience of the profession when they are carefully compiled as this one has been. There are many cases constantly arising in which for the time being a drug has slipped out of memory. Here is where a formulary comes in handy. And then it is a most useful between-times reminder. Picked up at odd moments and glanced through it will certainly add something to the mental storehouse, which will be of use later.

There is an apparent effort throughout to summarise the best therapeutics without regard to age, novelty or fads. The diseases are arranged in alphabetical order, and the best treatment is outlined according to the indications which are given in brief but comprehensive form. The Medical News Formulary is valuable because it is good, it is invaluable because it is safe. N. W. W.

